# Prevalence of Thyroid Dysfunction Among Depression Patients in a Tertiary Care Centre

**DOI:** 10.31729/jnma.5296

**Published:** 2020-09-30

**Authors:** Bikram Kafle, Bikram Khadka, Mohan Lal Tiwari

**Affiliations:** 1 Department of Psychiatry, Devdaha Medical College, Rupandehi, Nepal; 2 Department of Biochemistry, Devdaha Medical College, Rupandehi, Nepal; 3 Department of Medicine, Devdaha Medical College, Rupandehi, Nepal

**Keywords:** *depression*, *hyperthyroidism*, *hypothyroidism*

## Abstract

**Introduction:**

Patients with thyroid disorders are more prone to develop depressive symptoms and conversely depression may be accompanied by various subtle thyroid abnormalities. The aim of the study was to estimate the prevalence of thyroid dysfunction in depression.

**Methods:**

This is a descriptive cross-sectional study conducted at Devdaha Medical College and Research Institute employing a simple random sampling technique during the period of August 2019-January 2020. The research was approved by the Ethical Committee of the Institutional Review Board of Devdaha Medical College and Research Institute. The protocol approval number is 009/019. Data analysis was done in Statistical Package for the Social Sciences (Version 23). Results were presented as frequencies and percentages where required.

**Results:**

Among 263 patients with depression, 69 (26.2%) had abnormal thyroid status with most common being subclinical hypothyroidism 32 (12.2%), 13 (4.9%) overt hypothyroidism and 7 (2.7%) overt hyperthyroidism.

**Conclusions:**

The prevalence of thyroid dysfunction is high among patients with depression. We recommend to conduct routine thyroid function tests for all the patients with depression.

## INTRODUCTION

Thyroid dysfunction has been found to be associated with variety of neuropsychiatric disturbances, like depression, mania , acute psychosis, and cognitive disorders.^1^,^2^ Overt hypo and hyperthyroidism are associated with increased risk of depression.^3^ Commonly documented abnormalities are elevated thyroxine (fT4) levels, low tri-iodothyronine (fT3), blunted thyroid stimulating hormone (TSH) response to thyrotropin-releasing harmone stimulation , and loss of the nocturnal TSH rise.^4^ The annual incidence of hyperthyroidism was also higher in patients with depression than in general population.^5^

Few studies have examined the prevalence of thyroid dysfunction in major depressive patients in Nepal. In a Neplease study , prevalence of thyroid dysfunction in depressive patients, found that 21% of depressive patients had abnormal thyroid function tests.^6^

The aim of the study is to find out the prevalence of thyroid dysfunction in the newly diagnosed depressed patient.

## METHODS

We conducted a descriptive cross-sectional study at Devdaha Medical College and Research Institute employing a simple random sampling technique during the period of August 2019-January 2020. The research was approved by the Ethical Committee of the Institutional Review Board of Devdaha Medical College and Research Institute. The protocol approval number is 009/019. The sample size of the study was calculated taking reference to the previous published study which reported 22% prevalence of thyroid disorder among depressive patients^7^ using the formula,

$$\begin{array}{ll} \text{n} & \text{= }{\text{Z}}^{\text{2}}\times \text{p}\times (1-\text{p)}/{\text{e}}^{\text{2}}\\ & \text{= }{\left(1.96\right)}^{\text{2}}\times (0.22)\times (0.78)/{\left(0.05\right)}^{\text{2}}\\ & \text{= }263 \end{array}$$

where,

n = sample size
Z = 1.96 at 95% CI.
p = prevalence of thyroid disorder among depressive^7^
e = margin of error (5%)

We enrolled 263 patients who attended the Psychiatry OPD of Devdaha Medical College and were diagnosed with depression.

Inclusion criteria were patients with age >15 and those who agreed to join the research. Exclusion criteria included those who were not able to give the information because of their severity, known thyroid disease in past, substance use, medical comorbidites like hypertension and diabetes and pregnant patients. A self designed structured proforma was devised to obtain the socio-demographic characteristics of the study population.

Depression was diagnosed by the Psychiatrist. Diagnosis and grading as mild, moderate and severe was done on the basis of ICD-10 DCR as developed by the division of Mental Health of the World Health Organisation (WHO,1992). As a rating scale Hamilton Rating Scale for Depression (HAM-D) was used for comparision with the clinical diagnosis of depression.

Five milliliter of venous blood sample at 8:00 am (to avoid the influence of circadian rhythm) was collected in a gel tubes. Blood samples were centrifuged at 3000 RPM for 10 minutes for separation of serum and stored at -20 degree celsius till get analysed. Serum levels of free T3, free T4, and TSH were determined with Electro-Chemiluminescence technique. Patients with thyroid function tests (TFT) reports from only Devdaha Medical College Laboratory were included to avoid bias. The normal values were as follows: free T3: 2.30-4.20 pg/ml; free T4: 0.89-1.76 ng/dl; TSH: 0.55-4.78 microIU/ml. Thyroid dysfunction was diagnosed by level of free T3, free T4 and TSH as follows. Subclinical hypothyroidism as normal fT3, normal fT4, and elevated TSH. Subclinical hyperthyroidism as normal fT3, normal fT4 and low TSH. Overt hypothyroidism as decreased fT3, decreased fT4, and elevated TSH, and Overt hyperthyroidism as elevated fT3, elevated fT4, and decreased TSH.

Data were analyzed using SPSS version 21 (Chicago, Illinois, USA). Descriptive analysis was performed and mean , median and standard deviation (SD) calculated. Data were explained in percentages.

## RESULTS

Mean age of the patient was 38.29±14.24 SD. Majority 143 (54.4%) of the patients were in age group 25-45. Out of total cases 164 (62.4%) were female, 220 (83.7%) were married, 107 (40.7%) had completed their secondary level of schooling. Majority of the cases 111 (42.2%) were homemaker. Regarding religion 246 (93.5%) cases were Hindu (Table 1).

**Table 1:** Comparison of socio-demographic variables among normal and abnormal thyroid status patients. n = 263

Variables		Normal thyroid status	Abnormal thyroid status	Total n (%)
Age group (years)	15-24	36 (13.68)	8 (3.04)	44 (16.7)
	25-34	64 (24.33)	13 (4.94)	77 (29.3)
	35-44	47 (17.87)	19 (7.22)	65 (25.1)
	45-54	20 (7.60)	12 (4.56)	32 (12.2)
	55-64	18 (6.84)	11 (4.18)	29 (11.0)
	65 and above	9 (3.42)	6 (2.28)	15 (5.7)
Sex	Male	82 (31.17)	17 (6.46)	99 (37.6)
	Female	112 (42.58)	52 (19.77)	164 (62.4)
Marital status	Single	24 (9.12)	6 (2.28)	30 (11.4)
	Married	158 (60.07)	62 (23.57)	220 (83.7)
	Seprated /widow	12 (4.56)	1 (0.38)	13 (4.9)
Educational status	Illiterate	25 (9.50)	18 (6.84)	43 (16.3)
	Primary	23 (8.74)	16 (6.08)	39 (14.8)
	Secondary	85 (32.31)	22 (8.36)	107 (40.70)
	Intermediate	39 (14.82)	10 (3.80)	49 (18.6)
	University	22 (8.36)	3 (1.14)	25 (9.5)
Religion	Hindu	181 (68.82)	65 (24.71)	256 (93.5)
	Buddhist	11 (4.18)	4 (1.52)	15 (5.7)
	Others	2 (0.76)	0	2 (0.8)
Occupation	Unemployed	15 (5.70)	4 (1.52)	19 (7.2)
	Homemaker	73 (27.75)	38 (14.44)	111 (42.2)
	Agriculture	15 (5.70)	8 (3.04)	23 (8.7)
	Service	56 (21.29)	9 (3.42)	65 (24.7)
	Business	22 (8.36)	6 (2.28)	28 (10.6)
	Student	13 (4.94)	4 (10.52)	17 (15.46)

According to ICD-10 DCR based diagnosis of depression, patients with moderate depression were predominant 206 (78.3%), followed by severe depression 44 (18.3%) and mild depression 9 (3.4%) (Table 2).

**Table 2:** Diagnosis of depression according to ICD-10 DCR and its association with thyroid status. n = 263

Diagnostic tool	Types of depression	n (%)	Normal thyroid status n (%)	Abnormal thyroid status n (%)
ICD-10	Mid depression	9 (3.4)	7 (2.66)	2 (0.76)
	Moderate depression	206 (78.3)	154 (58.54)	52 (19.77)
	Severe depression	48 (18.25)	33 (12.54)	15 (5.70)

Categorization of patients on basis of thyroid status was done which shows only 69 (26.2%) of the depressed participants has had thyroid disorder of which Subclinical hypothyroidism was major thyroid disorder 32 (12.2 %) and Inappropriate TSH secretions 5 (1.9%) was least (Table 3).

**Table 3:** Distribution of patients on basis of thyroid status. n = 263

Status of thyroid	N (%)
Normal	194 (73.8)
Hypothyroidism	13 (4.9)
Subclinical hypothyroidism	32 (12.2)
Subclinical hyperthyroidism	4 (1.5)
Overt hyperthyroidism	7 (2.7)
Inappropriate TSH secretions	5 (1.9)
Secondary hypothyroidism	8 (3)
Total	263 (100)

The mean value of HDRS score was 23.04±7.65 SD. Mean of f T4 1.27±0.55 SD, f T3 2.88±1.06 and median value of TSH was 2.34 (Table 4).

**Table 4:** Mean, median and SD of thyroid hormones and HDRS score.

Descriptives	Age	TSH(mIU/ml)	f T4(ng/dl)	fT3(pg/ml)	HDRS score
Mean±SD	38.29±14.24	4.70±14.73	1.27±0.55	2.88±1.06	23.04±7.65
Median	35	2.34	1.23	2.69	21

## DISCUSSION

The prevalence of thyroid dysfunction among depressive patients was found high in this present study. Among 263 patients with depression, slightly more than one fourth 26.2% had abnormal thyroid status. This findings of our study is in accordance with the study conducted by Charnsil, Ojha, Das and Loosen which showed 22.1% , 21%, 19.34% and 25% respectively.^6–9^ It was seen that 4.9% of patient had overt hypothyroidism, 12.2% had subclinical hypothyroidism and 6.1% had hyperthyroidism. This is in concordance with study conducted in Nepal which showed 11.4% having subclinical hypothyroidism and 4.3% had overt hypothyroidism.^6^ Our findings differed with findings from other parts of the globe which showed lower prevalence of subclinical hypothyroidism.^7,10^

The mean age of our study participants was 38.29±14.24. This can be explained by the fact that the common age group for depression is middle age with 50% occurring in the 20 to 50 years age group. We next analysed that there was a huge gender gap in depression of our study participants. More than half 62.4% of study population of this study were female. The more percentage of females with depression could be due to the prevalence of higher depression in female than male as well as some socioeconomic and family-related factors moderate the relationship between gender and depression.^11^ Community surveys of symptoms of anxiety and depression have generally shown female: male ratio of 2:1, a finding supported by the large scale epidemiological catchment area (ECA) survey carried on US.^12^

Other major findings of our study is that moderate depression is the most common type of depression with 78.3% of patients, while 18.3% patients had severe depression and 3.4% were found to suffer from mild depression. Such a huge difference in the prevalence of the type of depression can be explained by the fact that mildly depressed patient usually do not seek medical help while severely depressed people usually go to emergency for help. These findings of our study are in concordance with the prevailing literature that it is mainly the moderate depression that are mostly diagnosed in outpatient basis.

Similarly study conducted in Nepal showed that fT3 and fT4 levels were found to be significantly raised in the moderate depression compared to the healthy controls.^8^ This difference in results might be because the onset of thyroid dysfunction in our group may have differed from other's studies.

Our study has some notable strength. First it has explored a new area of hospital based study among depressed patients. Secondly, this study advices early detection of thyroid disorders among depressive patients for better health outcomes.

The limitation of our study was that it was a hospital based cross-sectional study with limited sample size so this limits the demonstration of the effects of the thyroid dysfunction had over the course of the severity of depression and cannot be generalized unless repeated in the community.

## CONCLUSIONS

The outcome of the study indicates high prevalence of thyroid disorder among depressive patients. Findings of our study emphasize in routine evaluation of thyroid hormone in depressive patients. However, a population based nationwide survey is recommended to reflect the actual epidemiology of thyroid disorder among depressive patients in Nepal as no such studies has been carried out so far.

## Conflict of Interest

**None.**
